# Microbial biosorbent for remediation of dyes and heavy metals pollution: A green strategy for sustainable environment

**DOI:** 10.3389/fmicb.2023.1168954

**Published:** 2023-04-03

**Authors:** Manikant Tripathi, Pankaj Singh, Ranjan Singh, Saroj Bala, Neelam Pathak, Sangram Singh, Rajveer Singh Chauhan, Pradeep Kumar Singh

**Affiliations:** ^1^Biotechnology Program, Dr. Rammanohar Lohia Avadh University, Ayodhya, Uttar Pradesh, India; ^2^Department of Microbiology, Dr. Rammanohar Lohia Avadh University, Ayodhya, Uttar Pradesh, India; ^3^Department of Microbiology, Punjab Agricultural University, Ludhiana, Punjab, India; ^4^Department of Biochemistry, Dr. Rammanohar Lohia Avadh University, Ayodhya, Uttar Pradesh, India; ^5^Department of Botany, Deen Dayal Upadhyaya Gorakhpur University, Gorakhpur, Uttar Pradesh, India

**Keywords:** biosorbent, dyes, genetic engineering, heavy metals, pollution, toxicity

## Abstract

Toxic wastes like heavy metals and dyes are released into the environment as a direct result of industrialization and technological progress. The biosorption of contaminants utilizes a variety of biomaterials. Biosorbents can adsorb toxic pollutants on their surface through various mechanisms like complexation, precipitation, etc. The quantity of sorption sites that are accessible on the surface of the biosorbent affects its effectiveness. Biosorption’s low cost, high efficiency, lack of nutrient requirements, and ability to regenerate the biosorbent are its main advantages over other treatment methods. Optimization of environmental conditions like temperature, pH, nutrient availability, and other factors is a prerequisite to achieving optimal biosorbent performance. Recent strategies include nanomaterials, genetic engineering, and biofilm-based remediation for various types of pollutants. The removal of hazardous dyes and heavy metals from wastewater using biosorbents is a strategy that is both efficient and sustainable. This review provides a perspective on the existing literature and brings it up-to-date by including the latest research and findings in the field.

## 1. Introduction

Technological advancements in industry, agriculture, and other anthropogenic activities such as battery production, pesticides, alloy production, phosphate fertilizer, photographic materials, sewage irrigation, tannery and textile wastes, and dyes industries have wreaked havoc on our ecosystem due to the discharge of massive amounts of toxic metals, hazardous waste such as dyes and metalloids, and organic contaminants (Dixit et al., 2015; Ayangbenro and Babalola, 2017; Garg and Tripathi, 2017; Garg and Chopra, 2022; Kinigopoulou et al., 2022; Pham et al., 2022; Saravanan et al., 2023). Naturally available heavy metals are tightly bound to the soil and cannot impact the life of a living being. Contamination of the ecosystem with these non-degraded heavy metals, dyes, and metalloids beyond the recommended limit is creating serious global health concerns and having a detrimental effect on all life forms (Yin et al., 2019; Elgarahy et al., 2021). Due to their non-degradative properties, most heavy metals persist for a long time in the environment, which causes bioaccumulation and toxicity. The toxicity of heavy metals and dyes depends on the concentration exposed to organisms, duration of exposure, absorbed dose, and routes of exposure (Mani and Kumar, 2014). Bioassimilation is the process by which organisms absorb particular nutrients, substances, or molecules from their environment into their own biological tissues. Bacteria and other microbes can bioassimilate (Nikolaivits et al., 2021). It is a crucial process in numerous biological systems, with both positive and negative effects on species and ecosystems (El-Sherif et al., 2022). Understanding the mechanisms and the consequences of bioassimilation is crucial for maintaining and preserving the health of biological systems.

Heavy metal and dye-contaminated sites are cleaned up using traditional wastewater treatment methods like chemical precipitation, ion exchange, adsorption, evaporative recovery, chemical oxidation or reduction reactions, reverse osmosis, electrolytic recovery, and sludge filtration (Tripathi and Singh, 2022). Two major groups of water pollutants, i.e., dyes and heavy metals, are contaminating the water due to the direct discharge of effluent from the pharmaceutical, paper and pulp, textile and dyeing industries, food processing, leather tanning, electroplating, and mining industries (Chowdhary et al., 2020; Munjur et al., 2020). Heavy metals and dyes contaminate the soil and water bodies and affect plant and microbial growth and productivity. So, there is a need to find out some safe technologies by which we can reduce the availability of heavy metals and dyes in the open environment (Singh et al., 2022a). Ancient writings showed that bacteria, cyanobacteria, microbial consortiums, fungi, yeasts, and plants have a lot of potential to get rid of metals and dyes through different processes. The mechanisms present in microorganisms to detoxify heavy metals and dyes can be used to design the bio-treatment in the bioremediation treatment process. Plant-based removal or detoxification of toxic metals and dyes also uses microbe-based strategies for successful decontamination of polluted sites. The bioremediation technique utilizes the inherent biological mechanisms of microorganisms and plants for the removal of heavy metals and dyes from polluted environments (Akcil et al., 2015; Ojuederie and Babalola, 2017; Tripathi and Singh, 2022).

Microbial remediation uses mechanisms of microorganisms such as bioaccumulation, biosorption, and biotransformation for the removal of heavy metals and dyes from polluted environments (Garg and Tripathi, 2017; Filote et al., 2021; Tripathi and Singh, 2022). These methods provide a better alternative and are more effective than physical and chemical techniques to remediate heavy metals. Microbial cells have several protection mechanisms against heavy metal toxicity, like active efflux of metal ions, reduction of metal ions, etc. (Choudhury and Srivastava, 2001; Garg and Chopra, 2022). It is well known that industrial dyes are dangerous, which makes them common contaminants (Garg and Tripathi, 2017; Bala et al., 2022). Dyeing is a common practice in many manufacturing sectors; this includes textiles, paper, plastics, leather, and many more. There are a number of techniques that have been proven to be effective at removing dye (Elgarahy et al., 2021; Tripathi et al., 2021, 2023). The cultivation of several microbial strains for use in biological treatment has been envisioned as a potentially lucrative solution (Yusuf et al., 2021).

**Figure 1:**
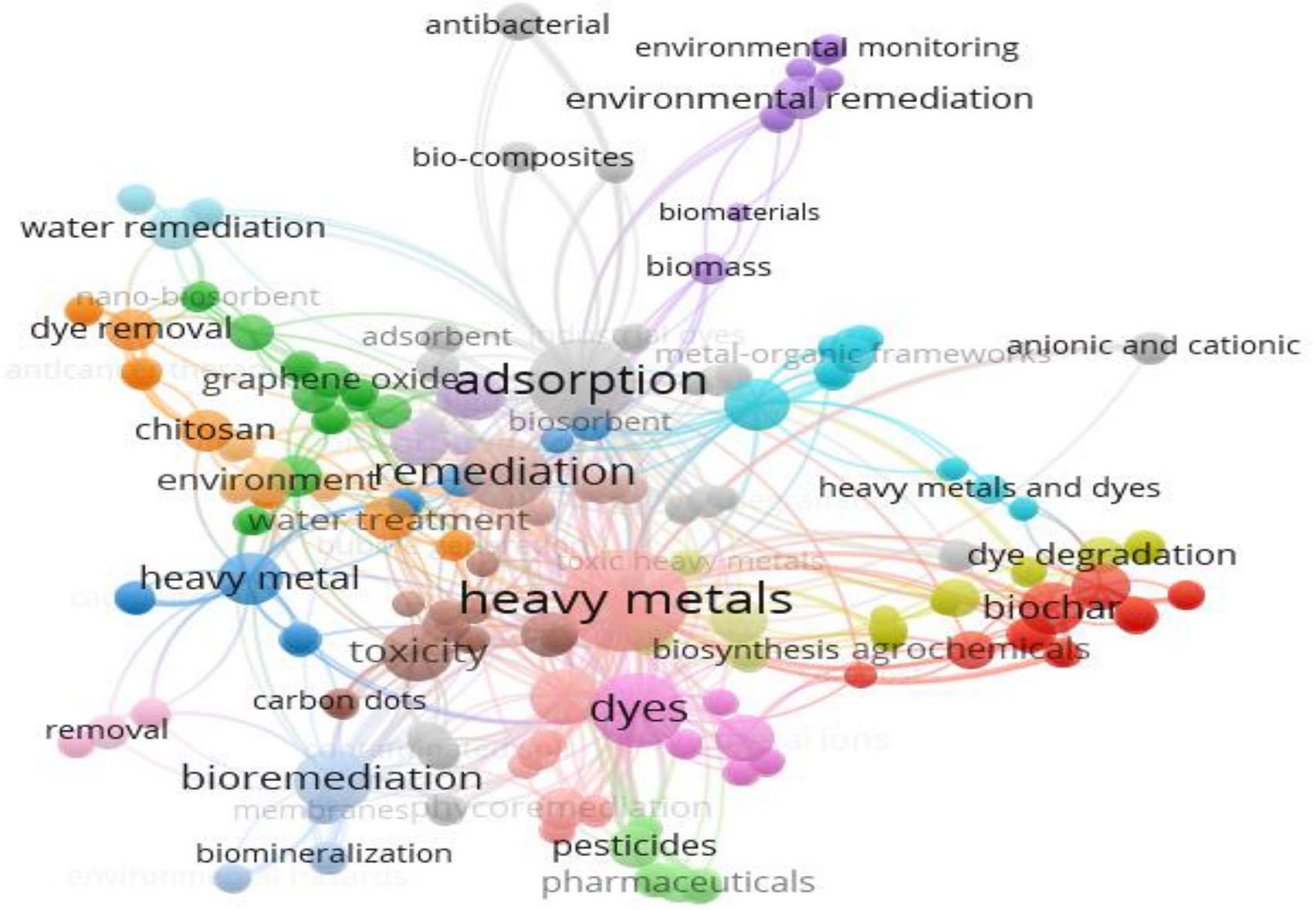
Network visualization of most frequently used keywords over time, as well as a clustering of the keywords’ citation networks.

Industrial effluent dye and heavy metal removal with microbial biosorbents seem promising. Microbial biosorbents can remediate these pollutants; however, there are still research gaps. Microbial biosorbents require strain selection, growing conditions, and immobilization. Optimizing these parameters increases biosorption efficiency (Nguyen et al., 2022). Microbial biosorbents have been studied mostly in labs. These experiments must be scaled up to assess the viability of utilizing microbial biosorbents in large-scale industrial applications. Industrial uses of microbial biosorbents depend on their long-term stability (Younas et al., 2021). Microbial biosorbent stability and reusability need further study. For a better understanding of microbial biosorbent for the remediation of dyes and heavy metal pollution, a graphical mapping of the keywords co-occurrence and co-authorship was made using the noncommercial visualization of similarities (VOS) viewer VOS viewer 1.6.18.^1^ The quantity of published work mainly from 2018 to 2023 is a powerful indicator of future paths of study. A keyword list can be used to classify various academic disciplines. Figure 1 shows a network visualization map of the most-cited keywords for the past few years.

Genetically modified microorganisms will have altered cells with efficient adsorption capacity and specificity for target metals (Wu et al., 2021; Rafeeq et al., 2023). The use of microbial fuel cells (MFC) and biofilm-based treatment can also be applied. Many parameters such as nutritional status, pH, temperature, chemical composition of heavy metals, moisture, and redox potential influence the bioremediation efficiency (Shukla et al., 2013). This review discusses the origin and harmful consequences of toxic metals and dye pollutants, eco-friendly microorganism-based and recent technologies for treating dyes and heavy metal-contaminated surroundings, and future prospects.

## 2. Sources and hazardous effects

A number of industries release heavy metals like chromium, lead, mercury, and others into the environment. According to Tripathi et al. (2018), heavy metals pose a significant risk to human health. On the other hand, dyes are the aromatic color preparations used in textile, paper, leather, food, cosmetics, tanneries, pharmaceutical industries, etc. (Shindhal et al., 2021; Singh et al., 2021a). In a study, Gita et al. (2017) suggested that synthetic dyes are made from petrochemical compounds and can be sold as powder, paste, liquid, or granules. Fabrics color quickly and evenly, offer a wide range of color shades, stay stable in the face of many outside factors, and use little energy (Hossen et al., 2019). So, most synthetic dyes are toxic when they are dumped into the biosphere without being cleaned or only partially cleaned (Ito et al., 2016; Singh, 2017).

Textile wastewater has a high biological and chemical oxygen demand, a high pH, and it also possesses organic and inorganic pollutants including heavy metals, chlorinated compounds, chromium, pigments, etc. (Donkadokula et al., 2020; Singh et al., 2021a,b). These contaminants have been held accountable for the pollution of local landfills and agricultural fields, which reduces plant growth by causing oxidative stress and lowering photosynthesis and CO_2_ assimilation rates, especially in developing countries (Vikrant et al., 2018). The damage was also caused by the dark color and high turbidity of the wastewater, which diminished aquatic photosynthesis (Donkadokula et al., 2020). The main contributors to heavy metals are listed below in Table 1.

**Table 1:** Sources and toxic impacts of heavy metals.

Pollutants	Sources	Hazardous effects	References
Mercury	Mining, paper and pulp, electrical equipment, cosmetics, coal power plants, cement, and pesticides	Minamata disease, abdominal pain, paralysis, and loss of appetite	Sharma et al. (2019)
Cadmium	Coal, batteries, nuclear and coal power plant, ceramics	Itai-Itai disease, lung fibrosis, dyspnea	Genchi et al. (2020) and Abbas et al. (2018)
Lead	Mining, coal, automobiles, paper dyeing, petrochemicals	Dyslexia, learning disability, mental retardation, anemia, and muscle and joint pain	Nag and Cummins (2022)
Chromium	Thermal power plants, leather tanning, mining fertilizers, textiles, and photography.	Sinus cancer, allergies, bronchial asthma, lung tumors	Sharma et al. (2022)
Uranium	Mining	Cancer	Balaram et al. (2022)
Zinc	Distilleries, phosphate fertilizers, and pharmaceuticals	Short-term illness, “metal fume fever,” and restlessness, Acrodermatitis enteropathica	Cao et al. (2022)
Nickel	Power plants, mining, automobile electroplating, coal, phosphate fertilizers,	Dermatitis, chronic bronchitis, cancer of the lungs	Abbas et al., 2014, Bharti and Sharma (2022), and Munir et al. (2022)
Copper	Electronic wastes	Wilson disease, long term exposes cause irritation to nose, eyes and headache	
Arsenic	Groundwater contamination by industrial pollutants	Cancer and skin lesions	
Chromates	Tanneries, paints, and corrosion inhibitors	Skin ulcers, blood systems, and brain damage	
Beryllium	Aerospace industry, ceramic parts	Acute berylliosis, lung cancer, heart, and lung toxicity	

## 3. Microbial biosorbent: A tool for dyes and heavy metals clean-up

Biosorption is a subfield of sustainable development that focuses on application. It is regarded as an eco-friendly, economical, and effective water treatment method. In accordance with various government regulations, it lowers the concentration of various water contaminants to acceptable levels. This eco-friendly protocol aligns with the concepts of green chemistry. The concept of biosorption as a multifaceted, effective process has evolved in recent years. It is regarded as an admirable alternative to conventional wastewater treatment technology. Sorption is primarily defined as a physico-chemical phenomenon involving the concentration of sorbate molecules on the surface of another substance. Adsorption is a physical interaction between sorbate and sorbent that produces a sorbent-sorbate contact (Nisa et al., 2022). Biosorption is a passive, metabolically independent process covering all sorbate-biological matrix contact features (Fomina and Gadd, 2014). Microbial biosorbents remove xenobiotic compounds from contaminated environments in a cost-effective and environmentally friendly way. Synthetic xenobiotic compounds can harm humans and the environment.

**Figure 2:**
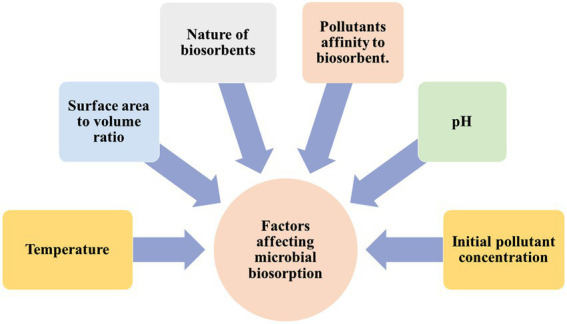
Factors for effective biosorption of pollutants.

Microbial biosorbents adsorb or absorb xenobiotic compounds from water or soil (Kour et al., 2021). Cell walls, exopolysaccharides, and extracellular proteins from microorganisms like bacteria, fungi, algae, and yeasts make up these materials. Microbial biosorbents remove xenobiotic compounds better than chemical or physical separation methods. Biodegradable, renewable, and inexpensive, microbial biosorbents (Adewuyi, 2020). They can also be customized to specific pollutants and used *in situ*, saving money on transportation and disposal of contaminated materials. Microbial biosorbents aim to reduce xenobiotic compound contamination while protecting the environment and human health (Bradu et al., 2022). Figure 2 presents some of the various elements that can have an effect on microbial biosorbent. It plays a significant role in numerous naturally occurring processes across various scientific disciplines. Several studies have demonstrated that numerous organisms, including prokaryotes and eukaryotes, have varying natural capacities to biosorb hazardous heavy metal ions as well as toxic dyes (Datta et al., 2020). *Anoxybacillus rupiensis* TPH1 was identified from the Tattapani hot spring in Chhattisgarh, India. This strain has the potential to be applied to the bioremediation of heavy metals and azo dyes (Mishra et al., 2023).

Toxicity activates numerous resistance mechanisms. The creation of metal-binding peptides like metallothioneins, the development of protein transporters of ligand–metal complexes from the cytoplasm to vacuoles, and the efflux of metal ions by cell wall ion channels are some of these methods. Plasmids are often used by bacteria because of their ability to encode tolerance mechanisms (Kumar et al., 2019). A number of studies have been carried out with the intention of enhancing the resistance and/or the capacity of microorganisms to collect heavy metal ions. *Bacillus* and P*seudomonas* sp. were used as potential biosorbents for the effective removal of toxic heavy metals from the environment (Ghosh et al., 2022). *Bacillus subtilis* was able to biosorb a maximum of 96% of chromium at a concentration of 25 g mL^−1^, whereas *P. aeruginosa* was able to biosorb a maximum of 91.0% of metal at a concentration of 400 g Cd mL^−1^ (Rizvi et al., 2020).

Most dye-containing wastewater is treated biologically through aerobic, anaerobic, or a combination of aerobic and anaerobic biodegradation. This is because it is cheap and does not make any harmful byproducts. A wide range of microorganisms were used to decolorize dye and mineralize it (Kapoor et al., 2021). Garg and Tripathi (2017) reported that different microorganisms could be used to get rid of dyes contamination from polluted sites. They have a tremendous capacity for taking in pollutants, regenerating themselves, and costing very little to run. Both pure and mixed bacterial cultures can use a wide variety of carbon or nitrogen sources (Shabir et al., 2022). There have also been a few reports of possible strategies for the biosorption of color chemicals by bacterial biomass (Ellafi et al., 2023). The ability of bacterial biomass to bind dye particles is facilitated by the presence of hydroxyl, carboxyl, amino, and phosphate groups in the peptidoglycan layer of the cell wall. Due to the fact that biosorption is a metabolically autonomous process, no nutrients are required to support bacterial cell growth. *Saccharomyces pastorianus* was immobilized using a straightforward entrapment method and then microencapsulated in alginate. The biosorption behavior of this immobilized strain of yeast was examined in relation to an organic pollutant known as cationic dye (Blaga et al., 2022).

## 4. Mechanism of biosorption

**Figure 3:**
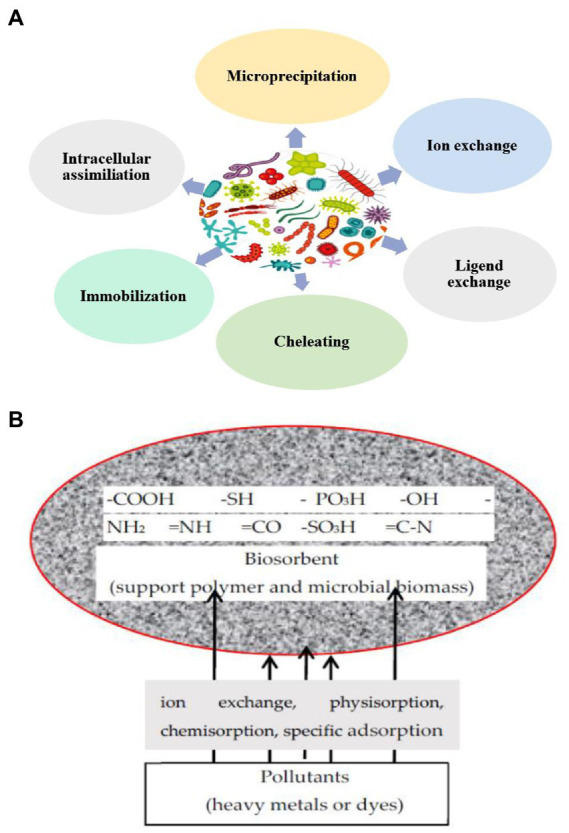
**(A)** Different ways to remediate environmental pollutants through biosorption process. **(B)** The biosorption mechanism of dyes and metals pollutants. **B** is reprinted from Blaga et al. (2021) and is an open-access article (Copyright © 2021 by authors) distributed under the terms and conditions of the Creative Commons Attribution (CC BY) license.

Biosorption mechanistic investigations of various water contaminants are essential for evaluating their control effectiveness. They are advantageous for optimizing the conditions of the removal procedure. There are several mechanisms through which biosorbent materials remediate environmental pollutants (Figure 3A). Biosorption uses microbial cells or biomolecules to remove pollutants from water or wastewater. Biosorption involves adsorption, diffusion, and binding. Microbial cells or biomolecules on the biosorbent attract aqueous phase pollutants during adsorption (Figure 3B). Physical or chemical interactions may bind pollutants to biosorbent functional groups like carboxyl, amino, or hydroxyl groups (Adewuyi, 2020). Adsorbed pollutants may disperse within the biosorbent and find more binding sites. Ion exchange, complexation, chelation, and electrostatic attraction can bind pollutants to biosorbents. The biosorbent’s surface area, surface chemistry, aqueous phase pollutants, pH, and temperature affect biosorption efficiency. Desorption or heat or chemical treatment can renew the biosorbent after biosorption. Biosorbent can be reused (Singh et al., 2022b). Biosorption is a potential method for removing toxic heavy metals and dyes from aquatic environment.

## 5. Recent strategies for pollutants remediation

Plants or microbes are used in bioremediation to treat polluted environments with xenobiotics and harmful heavy metals by converting them into nontoxic compounds (Bala et al., 2022; Tripathi and Singh, 2022). Improved bioremediation results can be achieved by a number of different methods, the choice of which is based on the level of pollution present in the immediate area. Bioremediation can be applied *in-situ* or *ex-situ*, whereas the *in-situ* bioremediation methods are more cost-effective and cause less pollution to be released into the environment (Rafeeq et al., 2023). Bioremediation using bacteria, fungi, yeasts, and algae is being studied. These microbes acted as toxic pollutant biosorbents (Kour et al., 2021). Biological methods (using bacteria, algae, fungi, and plants) have also been used successfully to treat dye-containing wastewater, but they are inefficient and risky (Singh et al., 2021b). In view of this, some other recent strategies are required for the remediation of textile dyes that are low cost and highly efficient.

### 5.1. Nanoparticle based

The use of nanoparticles (NPs) to clean up textile dyes is very promising right now, since NPs are better at cleaning up dyes and are better for the environment than other methods with applications in various fields. They are nanoscale materials and have huge potential for removing textile dyes (Chen et al., 2009). NPs are the tiniest structural arrangements with precision, intelligibility, and cost-effectiveness (Singh et al., 2019). Nanoparticles for bioremediation of hazardous textile dyes can be prepared by a variety of methods (Chandhru et al., 2020). The structure, shape, size, pH, dye concentration, and purity of nanomaterials play an important role in the process of decolorization of wastewater (Muthukumaran et al., 2016). They are mostly applied directly to wastewater for the adsorption of dyes on their surface (Fabryanty et al., 2017). They might also be used in membranes, filters, and carbon nanotubes for bioremediation of discharged textile wastewater (Kumari et al., 2017). Several reports were available that demonstrate dye degradation using nanocomposites such as polyaniline/SiO (Tanzifi et al., 2018), graphene oxide/zinc oxide (GO/ZnO; Durmus et al., 2019), and RGO-Ni (Das et al., 2019). In an investigation, Umar et al. (2021) used Pd-Ni bimetallic nanoparticles for the successful removal of the dye Acid Orange 8. In some studies, NPs were utilized for the removal of heavy metals, microbes, and oil from wastewater (Bayazit et al., 2019; Kausar et al., 2022). A variety of nanomaterials, including TiO, ZnO, and metallic nanoparticles such as gold, silver, and iron, have been tested for the catalytic degradation of toxic dyes (Singh et al., 2019).

Cds/GO nanoparticles are a nanomaterial composed of cadmium sulfide (Cds) and graphene oxide. They are suitable for use in bioremediation due to their unique properties. GO is a highly oxidized form of graphene that has the ability to adsorb a wide variety of organic and inorganic compounds. Cds/GO nanoparticles can interact with xenobiotics in multiple ways when added to contaminated soil or water. First, Cds can absorb light and produce ROS, which are capable of degrading xenobiotics. Second, GO is capable of adsorbing xenobiotics and reducing their concentration in the environment (Gul and Ahmad, 2022). Cds/GO nanoparticles have been utilized to remove dyes from wastewater, degrade polycyclic aromatic hydrocarbons (PAHs) in soil, and remove pesticides from water (Safapour et al., 2022).

### 5.2. Through biofilms

A biofilm is an assemblage of microbes that normally originates in wet environments. Biofilm is produced from organic and inorganic substances that are generated from decaying material in wastewater. Biofilm-mediated degradation involves the enzymatic decomposition of various organic compounds and biosorption, during which elements convert from the liquid phase to the solid phase. Bacterial biofilms can remove dyes and achieve decolorization by using biosorption and degradation mechanisms (Watnick and Kolter, 2000). Bacteria, fungi, algae, and protozoa are also the organisms that form biofilms by generating an extracellular polymeric substance (EPS). EPS holds the microbial cells in biofilms together as they accumulate into three-dimensional assemblages that secrete glue-like material for attachment to any hard surface (Singh et al., 2019). Comparing fungal–bacterial biofilms to their individual cultures, Nigrosin disodium and Malachite Green dyes are more effectively degraded. In this experiment, *Trichoderma harzianum*, *Pseudomonas fluorescens*, and *Bacillus subtilis* produced a biofilm that had the highest dye removal ability for both Nigrosin disodium and Malachite Green dyes (Henagamage, 2019). Biofilm consortiums were also useful in the color removal, degradation, and detoxification of the azo dye Methyl Orange (Haque et al., 2021). Congo Red was removed by *Bacillus* sp. MH587030.1-derived polyurethane/polypropylene biofilm in a moving bed reactor (Sonwani et al., 2020). Biofilm-mediated textile wastewater treatment offers numerous advantages, like operational flexibility, low space requirements, a high biomass residence time, enhanced bioremediation of recalcitrant compounds, and the production of a small amount of sludge (Singh et al., 2019).

### 5.3. Genetic engineering: A bioremediation tool

Recently, genetic engineering has been shown to be an important tool for making dye removal bioremediation processes that are more effective and better for the environment. This has made a big change in this field. This process can be useful for the absolute bioremediation of pollutants, including textile dyes (Singh et al., 2019). It involves identification, isolation, cloning, and transferring genes that encode degradative enzymes. These enzymes enhance the bioremediation potential of native microbes. These engineered degraders, called super degrading microorganisms (Pereira and Alves, 2011), exist as engineered or hybrid strains (Kandelbauer and Guebitz, 2005).

Every microorganism has different potential for bioremediation of textile dyes. Genetically modified microorganisms (GMMs) can be produced by transferring genes from one species to another or by gene modification (Saxena et al., 2019; Kumar et al., 2020). The bacterium *Escherichia coli* that was engineered with the azoreductase enzyme gene of *Pseudomonas luteola* effectively degrades textile dyes (Chang et al., 2000). Similarly, Dixit and Garg (2018) transferred the azoreductase enzyme-coding gene azoK into *E. coli* from the bacterium *Klebsiella pneumonia* to achieve successful decolorization of the dye Methyl Orange. Ajaz et al. (2020) demonstrated the removal of Remazol Red dye with the help of the azoreductase gene. Although some ecological safety issues exist with GMMs, they can be overcome by utilizing the products, enzymes, or intact cells containing overexpressed enzymes (Singh et al., 2019).

## 6. Challenges, research gaps, future directions, and applications

For biosorbents to attain their full potential, additional research is required in a variety of fields. Sonication, freeze-drying, and the addition of new functional groups are just a few of the strategies that have been used to increase the sorption activity of biosorbents (Anderson et al., 2022). Extending the biosorption capacity of various biosorbents to remove heavy metals and dyes from aquatic environments should be a primary focus of future research. Very little work has been done to assess the risks to the environment and human health associated with using biosorbents for such pollution remediation (Elgarahy et al., 2021). Moreover, xenobiotics and pharmaceuticals are becoming new environmental concerns. They can enter the environment through air, water, or soil contamination and last long. They can survive in the environment and accumulate in the food chain. These contaminants pose health and environmental risks (Singh and Singh, 2020). Xenobiotics and pharmaceuticals can cause reproductive and developmental issues, hormonal imbalances, and cancer in humans and animals. Biosurfactants have the potential to be used in future directions due to their capacity to decrease surface tension and increase the solubility and bioavailability of hydrophobic substances (Parthipan et al., 2021). Biosurfactants improve microbial bioremediation of dyes and heavy metals. Textile, printing, and dyeing industries use synthetic organic dyes. Persistent, toxic, and polluting, Biosurfactants increase dye solubility and microbial degradation, accelerating pollutant removal. Biosurfactants improve dye-contaminated wastewater dispersion and flocculation and sedimentation. Heavy metals in the environment can cause health issues. Biosurfactants make heavy metals more soluble and mobilizable for microbial biodegradation (Shukla et al., 2019). Biosurfactants help form metal-mineral complexes that immobilize heavy metals and reduce their bioavailability. Biosurfactants increase dye and heavy metal solubility and bioavailability, improving microbial bioremediation. Sustainable and environmentally friendly dye and heavy metal remediation using biosurfactants is promising (Deb et al., 2020).

Most of the time, biosorption has been shown to be better at absorbing things than bioaccumulation. To increase microorganisms’ tolerance for the accumulation of heavy metals, scientists have developed microbial genetic engineering. To further enhance the physical and chemical stability, immobilization of bacterial biomass on a suitable carrier may also be addressed (Singh et al., 2022b; Tripathi and Singh, 2022). While the benefits of biosorption with immobilized bacterial biomass seem promising, there are limitations to this approach because the underlying mechanism of the process is not fully known. In addition to biotechnology and nanotechnology, there are a number of other methods that, when combined, could give cutting-edge bioengineering technologies.

Biosorption of heavy metals and bioremediation of dyes have demonstrated promising future applications in a variety of fields. Industrial wastewater containing a high concentration of heavy metals and dyes can be treated using biosorption technology (Chu and Phang, 2019). This technology can be used to eliminate these contaminants from wastewater prior to its release into the environment. Biosorption technology can be employed to remove heavy metals from soil, thereby enhancing soil quality and crop yield. This technology can also be used to treat heavy metal- and dye-contaminated agricultural wastewater (Singh et al., 2020). Using biosorption technology, contaminated soils and groundwater can be remedied. This technology can be used to remove heavy metals and dyes from polluted soil, thereby contributing to the restoration of the natural environment. Biosorption technology has the potential to become a sustainable and cost-effective method for removing dyes from a variety of sources (Danouche et al., 2021).

## 7. Conclusion

This review paper explores biosorbent technologies for remediating heavy metal and dye-contaminated waterbodies. Use of biosorbent technologies provides attractive features like being readily available, potentially efficient, and inexpensive. In the biosorption process, microbial cells utilize adsorption phenomena, which include ionic, chemical, and physical ones. A large number of functional groups on the surface of microbial cells attract metals and dye metabolites. It has been observed that the biosorbent technologies can remove heavy metals from polluted aqueous solutions and have high adsorption capacities for synthetic dyes. For the sustainable development of effective biosorption technologies, there is a need to explore biosorbent characterization, efficient mechanisms of heavy metal removal, operating conditions, and microbial growth.

## Author contributions

MT: conceptualization, writing-original draft, and reviewing, editing, and finalizing the manuscript. PS, RS, and SB: writing—original draft, reviewing and editing, and drawing figures and table. NP, SS, and RC: reviewing and editing. PKS: writing-original draft and reviewing, editing, and finalizing the manuscript. All authors contributed to the article and approved the submitted version.

## Conflict of interest

The authors declare that the research was conducted in the absence of any commercial or financial relationships that could be construed as a potential conflict of interest.

## Publisher’s note

All claims expressed in this article are solely those of the authors and do not necessarily represent those of their affiliated organizations, or those of the publisher, the editors and the reviewers. Any product that may be evaluated in this article, or claim that may be made by its manufacturer, is not guaranteed or endorsed by the publisher.

## Footnotes

^1^ https://www.vosviewer.org/
